# Influence of Laccase and Tyrosinase on the Antioxidant Capacity of Selected Phenolic Compounds on Human Cell Lines

**DOI:** 10.3390/molecules200917194

**Published:** 2015-09-18

**Authors:** Matthias Riebel, Andrea Sabel, Harald Claus, Petra Fronk, Ning Xia, Huige Li, Helmut König, Heinz Decker

**Affiliations:** 1 Institute for Molecular Biophysics, Johannes Gutenberg University of Mainz, Jakob-Welder-Weg, 26, 55128 Mainz, Germany; E-Mails: riebelm@uni-mainz.de (M.R.); petra.fronk@uni-mainz.de (P.F.); 2 Institute for Microbiology and Wine Research, Johannes Gutenberg University of Mainz, Johann-Joachim-Becher-Weg, 15, 55128 Mainz, Germany; E-Mails: sabel@uni-mainz.de (A.S.); hclaus@uni-mainz.de (H.C.); hkoenig@uni-mainz.de (H.K.); 3 Department of Pharmacology, Johannes Gutenberg University Medical Center of Mainz, Obere Zahlbacher Str. 67, 55131 Mainz, Germany; E-Mails: xianing@uni-mainz.de (N.X.); huigeli@uni-mainz.de (H.L.)

**Keywords:** polyphenols, tyrosinase, laccase, oxidation, antioxidant activity, DPPH•, cell cultures

## Abstract

Polyphenolic compounds affect the color, odor and taste of numerous food products of plant origin. In addition to the visual and gustatory properties, they serve as radical scavengers and have antioxidant effects. Polyphenols, especially resveratrol in red wine, have gained increasing scientific and public interest due to their presumptive beneficial impact on human health. Enzymatic oxidation of phenolic compounds takes place under the influence of polyphenol oxidases (PPO), including tyrosinase and laccase. Several studies have demonstrated the radical scavenger effect of plants, food products and individual polyphenols *in vitro*, but, apart from resveratrol, such impact has not been proved in physiological test systems. Furthermore, only a few data exist on the antioxidant capacities of the enzymatic oxidation products of phenolic compounds generated by PPO. We report here first results about the antioxidant effects of phenolic substances, before and after oxidation by fungal model tyrosinase and laccase. In general, the common chemical 2,2-diphenyl-1-picrylhydrazyl assay and the biological tests using two different types of cell cultures (monocytes and endothelial cells) delivered similar results. The phenols tested showed significant differences with respect to their antioxidant activity in all test systems. Their antioxidant capacities after enzymatic conversion decreased or increased depending on the individual PPO used.

## 1. Introduction

Polyphenols (*i.e.*, phenols with at least two hydroxyl groups linked to the aromatic ring) comprise a large and diverse group of secondary plant metabolites, such as flavanols and their proanthocyanidin oligomers, anthocyanins, hydroxylated stilbenes, flavonols and hydroxy cinnamic acids. They are responsible for the major organoleptic characteristics of plant-derived food and beverages, particularly color and taste properties [1,2]. Polyphenols provide potential benefits to human health by many biological effects, such as antioxidants, cardioprotectives, antimicrobials, antifungals, antidiabetics, neuroprotectives, anti-inflammatories and antitumorals [1]. Thus, they contribute to the prevention of various diseases associated with oxidative stress and age-related disorders, such as coronary heart disease, cancer and neurodegeneration. Most of these effects are attributed to their powerful antioxidant properties, being able to scavenge reactive oxygen species (ROS) and oxidative-generated free radicals (e.g., oxidized low density lipoprotein). Current studies also suggest their interaction with cellular functions at different molecular levels, such as gene expression, protein synthesis and enzyme activities [3,4,5,6,7]. *Trans*-resveratrol and its glycoside *trans*-piceid (also known as polydatin) have received a lot of research attention due to a large number of postulated health effects [7,8,9].

Nonenzymatic and enzymatic oxidation of phenolic components and subsequent browning is an economically relevant problem since it impairs the organoleptic and color properties of fruits, vegetables, juices and wine. Polyphenol oxidases (PPO), including tyrosinases and laccases, are responsible for enzymatic browning reactions [10,11,12,13]. These metalloenzymes containing copper are ubiquitously distributed in different organisms, including plants. Their role in plant metabolism is not clear, but they may be involved in lignification, pigmentation and resistance to pathogens and herbivores [14,15].

Tyrosinases belong to the type-3 copper protein family and catalyze the hydroxylation of various monophenols and their subsequent oxidation to the corresponding *o*-diquinones. Thus, they exhibit both monophenolase and diphenolase activity. Quinones are highly reactive components which self-polymerize nonenzymatically or react with other substances, often forming insoluble high molecular pigments, e.g., melanin [15,16,17,18,19,20,21].

Laccases are oxidases containing a multicopper center. Unlike tyrosinase, they exhibit no monophenolase activity, but use molecular oxygen to oxidize a broad spectrum of organic compounds by a radical mechanism. Similar to tyrosinases, polyphenols are converted into quinones that successively polymerize in nonenzymatic reactions to form high molecular weight compounds [22,23].

Even today, a deep knowledge of the reaction mechanism of PPO with many antioxidants remains unclear [24]. In addition, several different oxidation products may occur depending on the specificity of the PPO and reaction conditions.

The question arises whether the activities of PPO change the antioxidant capacity and other health promoting properties of phenolic compounds. To the best of our knowledge, the antioxidant activity of PPO-oxidized phenolic substrates has only been assessed in a few cases, e.g., for resveratrol [25] and ferulic acid [26], using chemical test systems. In our study, we investigated the influence of fungal model enzymes—tyrosinase from *Agaricus bisporus* and a laccase from *Polyporus pinisitus*—on a selection of different phenolics with regard to scavenger activity and antioxidant capacity in cell cultures.

## 2. Results and Discussion

We investigated how enzymatic oxidations alter the antioxidant capacity of phenolic compounds. For that reason, we tested the action of fungal enzymes—a tyrosinase from *Agaricus bisporus* and a laccase from *Polyporus pinisitus*—on a selection of phenolic substrates representing typical compounds of flavonoids, stilbenes, benzoic acids and cinnamic acids.

As shown in Table 1, both PPO oxidized all phenolic compounds investigated, although to different extents. The concentrations of tyrosinase and laccase utilized depended on their enzyme kinetics. In contrast to tyrosinase, the laccase showed a much higher activity and, thus, the concentration of laccase required to gain a conversion rate in a similar range was much lower.

**Table 1:** Activity of PPO with different phenolic substrates.

Substrate (5 mM)	O_2_ Consumption (mg/L min)	
	Tyrosinase	Laccase
Resveratrol	3.24	2.29
Polydatin	1.14	2.95
Coumaric acid	0.46	0.26
Caffeic acid	33.10	2.86
Ferulic acid	0.44	2.61
Gallic acid	0.92	1.64
Phlorizin	2.16	0.18

We used two different test systems for the evaluation of antioxidant activities. Firstly, the well-established 2,2-diphenyl-1-picrylhydrazyl (DPPH•) radical scavenging assay [27], which is a chemical *in vitro* test. It is based on H-atom transfer or single-electron transfer from a phenolic OH group to the stable colored radical DPPH•. This reaction was monitored colorimetrically.

Secondly, a newly developed sensitive biological test that is able to detect additional physiological effects was applied. The underlying principle may need some explanation here. Nitric oxide (NO) produced by the endothelial NO synthase is a crucial protective factor because of its antithrombotic, antihypertensive and antiatherosclerotic properties [9]. In the state of oxidative stress, enzymatic production of ROS exceeds the available antioxidant defense systems. Superoxide reacts with NO and leads to loss of NO bioactivity. The resulting peroxynitrite may cause dysfunction of the endothelial NO synthase and, thus, reduce NO production. The reduced bioavailability of vascular NO is the major mechanism of endothelial dysfunction observed in cardiovascular disease [28].

Circulating monocytes undergo phenotype transition to macrophages that accumulate lipid in atherosclerotic lesion formation. Both monocytes and macrophages play a critical role in early atherogenesis. Monocytes contribute to atherogenesis by altering the local redox state through nicotinamide adenine dinucleotide phosphate (NADPH) oxidase-mediated superoxide production [29].

Therefore, the reduction of superoxide levels in EA.hy 926 cells and THP1 cells suggests potential protective effects of the phenolic compounds from cardiovascular disease by prevention of oxidative stress. THP1 cells are a human monocytic cell line and produce superoxide via NADPH oxidase, which was activated by PKC stimulation with phorbol 12-myristate 13-acetate (PMA) [30]. In EA.hy 926 endothelial cells, we used 2,3-dimethoxy-1,4-naphthoquinone (DMNQ), a quinone compound that generates superoxide and hydrogen peroxide continuously through redox cycling [31]. Superoxide is monitored by a CL method in these test systems.

The reduction observed of the superoxide signal by the phenolic compounds in the cells used can be attributed to three possible mechanisms: (i) direct superoxide-scavenging effects of the phenolic compounds; (ii) stimulation of the endogenous antioxidant enzymes in the cells (*i.e.*, superoxide dismutases); or (iii) inhibition of NADPH oxidase or PKC by the compounds (in THP-1 cells).

**Figure 1:**
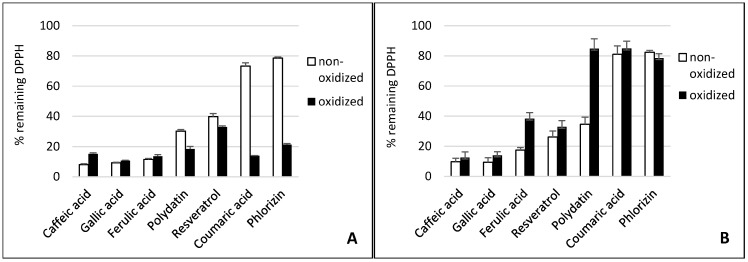
Effect of tyrosinase (**A**) and laccase (**B**) on the radical scavenging activity of phenolics compounds. The absorption of the colored 2,2-diphenyl-1-picrylhydrazyl (DPPH•) at 515 nm declines when reduced by an antioxidant. Substrates are lined in declining scavenging activity.

**Figure 2:**
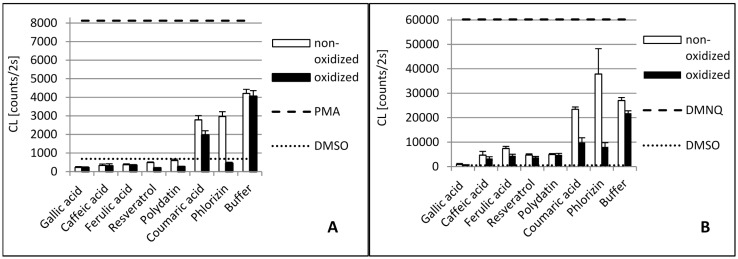
Antioxidant capacity of phenolic compounds as well as their **tyrosinase** oxidation products in THP1 (**A**) and EA.hy 926 (**B**) cells. In order to stimulate ROS production, phorbol 12-myristate 13-acetate (PMA) was added to THP1 cells and 2,3-dimethoxy-1,4-naphtoquinone (DMNQ) to EA.hy 926 cells, respectively. Due to interaction with ROS, added L-012 exhibits chemiluminescence (CL), which was detected. The PMA/DMNQ lines represent maximum ROS production by stimulated cells. The dimethyl sulfoxide (DMSO) line shows the ROS signal in cells without stimulation. Buffer: citrate buffer (0.1M, pH 5.0) without phenolic compounds.

**Figure 3:**
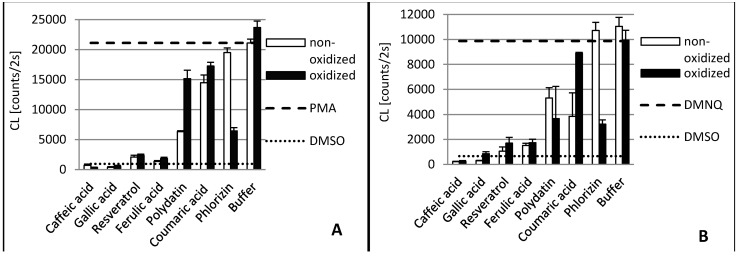
Antioxidant capacity of phenolic compounds as well as their **laccase** oxidation products in THP1 (**A**) and EA.hy 926 (**B**) cells. In order to stimulate ROS production PMA was added to THP1 cells and DMNQ to EA.hy 926 cells, respectively. Due to interaction with ROS, added L-012 exhibits chemiluminescence (CL), which was detected. The PMA/DMNQ lines represent maximum ROS production by stimulated cells. The DMSO line shows the ROS signal in cells without stimulation. Buffer: tartrate-malate buffer (30 mM, pH 3.5) without phenolics.

The antioxidant activity of phenolic compounds and enzymatic reaction products has been studied and compared in these different test systems. Figure 1 displays the scavenger activity of the phenolic compounds and their oxidation products against DPPH•. Figure 2 and Figure 3 contain the results of the antioxidant capacity of the same samples in cell cultures. Figures should be mentioned in a sequential order.

### 2.1. Gallic Acid and Caffeic Acid 

**Figure 4:**
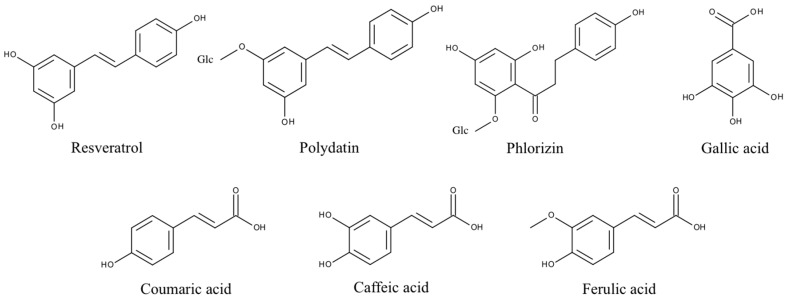
Structures of phenolic PPO substrates used in this study. Polydatin and phlorizin are glycosylated (Glc) polyphenols.

Gallic acid and caffeic acid reduced DPPH• immediately, thus showing the strongest scavenging effect (Figure 1). This can be explained by their chemical structure (Figure 4). Three adjacent hydroxyl groups in Gallic acid make them prone to H-atom transfer. Their relative position to each other results in strong stabilizing hydrogen-bonding interactions of generated phenoxy radicals. In addition, hydroxyl groups show a positive mesomeric effect (+M-effect), which further stabilizes the resulting radicals. Furthermore, +M-effects result in higher electron density in the delocalized π-system. This, in turn, weakens the bond dissociation energy of the O-H bond. Caffeic acid has only two adjacent hydroxyl groups. However, the acrylic acid group has a strong +M-effect, which explains the general high antioxidative potential of hydroxycinnamates.

Although laccase and tyrosinase are both able to oxidize Gallic acid, there was no significant difference between the oxidized and nonoxidized Gallic acid in the DPPH• assay and cell lines (see Figure 1, Figure 2 and Figure 3).

The tyrosinase and laccase oxidation products of caffeic acid showed a similar or slightly stronger antioxidative capacity than their educts across both cell lines. This was in contrast to the DPPH• results. Here, the tyrosinase oxidation products reduced DPPH• slightly less efficiently. This suggests that oxidation products of caffeic acid exert additional antioxidative effects in human cells besides direct radical scavenging. Laccase oxidation products of caffeic acid revealed the same scavenging effect as the educts.

### 2.2. Ferulic Acid

Ferulic acid differs from caffeic acid in a methylated hydroxyl group. In theory, this would reduce the antioxidative capacity, because only one hydroxyl group is available for H-atom donation. In addition, the +M-effect of a methoxy group is smaller than that of a hydroxyl group. However, stabilizing hydrogen bond interaction with the adjacent hydroxyl group is still possible. Nonetheless, the *chemical* scavenging effect of ferulic acid was comparable to caffeic acid. The treatment of ferulic acid with laccase led to a distinct decreased scavenger effect on DPPH• radicals. By contrast, dimerization of ferulic acid by a laccase from *Trametes versicolor* amplified antioxidant activity [26]. The difference in scavenging activity between ferulic acid and its tyrosinase oxidation products was negligible. Ferulic acid and its oxidation products also showed a high reduction of superoxide in the cell cultures.

### 2.3. p-Coumaric Acid

*p*-Coumaric acid differs from caffeic acid in having only one phenolic hydroxyl group. In addition, there is no hydrogen bond interaction possible that could stabilize emerging phenoxy radicals. This results in the very weak scavenging activity of *p*-coumaric acid. This agrees with previous findings that show monophenols to either lack or present less antiradical activity than diphenols [32,33]. *p*-Coumaric acid’s tyrosinase oxidation products, however, displayed a strong scavenging effect, comparable to those of caffeic acid. This can be explained by the monohydroxylation of *p-*coumaric acid in *ortho* position to the first hydroxyl group, resulting in the same oxidation products as caffeic acid. *P. pinisitus* laccase oxidized coumaric acid slightly (Table 1), but the oxidation products showed less scavenging activity than its educts.

### 2.4. Resveratrol and Polydatin

Because of the relative position of hydroxyl groups to each other, resveratrol and its monoglycoside polydatin lack the potential to form radical stabilizing intramolecular hydrogen bonds. Additionally, they have a relatively large delocalized π-system, which lowers their ionization potential. That is why resveratrol is one of the so-called “slow” antiradicals [34]. Regarding scavenging mechanisms, this makes them more prone to single-electron transfer. Resveratrol and polydatin showed a similar scavenging activity when reducing DPPH•. Both tyrosinase oxidation products revealed a slightly stronger scavenging effect than their respective educts. By contrast, the laccase oxidation products exerted a more reduced scavenging activity against DPPH• than their educts. Whereas oxidized resveratrol only showed a slightly reduced effect, the oxidized polydatin exerted almost no scavenging effect. The same could be seen in the cell culture where our results confirmed the reduction of superoxide levels by resveratrol. However, the oxidation products of resveratrol, which is the result of tyrosinase or laccase activity, showed similar antioxidative capacity, suggesting that the action of oxidizing enzymes did not alter the antioxidative effects of resveratrol with reference to THP1 and EA.hy 926 cells significantly. This confirms earlier *in vitro* studies showing that oxidation of resveratrol by fungal tyrosinase and laccases did not alter its antiradical capacity [25,35].

Polydatin, by contrast, showed a decreased effect after oxidation with laccase. An explanation could be that the antiradical activity of resveratrol is due to its so-called “*m*-diphenol core”, which might not be affected by PPO-catalyzed oxidation [25]. Blocking one of the hydroxyl groups in this *m*-diphenol core of resveratrol by a glycosidic residue, as in polydatin, could make the other hydroxyl group available for oxidation, which leads to a reduced antioxidant capacity.

### 2.5. Phlorizin

Whereas phlorizin showed only a weak scavenging activity, its tyrosinase oxidation products revealed a strong effect. Phlorizin has two unglycosylated hydroxyl groups in its A ring. No stabilizing hydrogen bond interaction for the resulting phenoxy radical is possible, because the hydroxyl groups are not adjacent. One additional hydroxyl group is located in the B ring. *ortho*-hydroxylation in this ring by tyrosinase might explain the much higher scavenging activity of phlorizin oxidation products. Even though no distinct influence of laccase on the scavenging effect of Phlorizin was observed in the DPPH• assay it was the only substance with improved antioxidative capacity when oxidized by laccase.

This effect could only be seen in both cell cultures. This manifests the suggestion that additional antioxidative mechanisms proceed in cells beyond the scavenging effect of superoxide.

To the best of our knowledge, no studies have been performed concerning the influence of different phenolic compounds on the ROS in the human cell lines we used. By contrast, several studies with different chemical *in vitro* assays have been performed to study the antioxidant potential of phenolic compounds [27]. It has to be mentioned that it is difficult to compare the results of our study with those of other investigations. There are many differences in the methods performed, concentrations of phenols used, radicals and even a wide range of possible evaluations of the results. Nevertheless, a lot of studies have shown that caffeic, Gallic and ferulic acid have a high potential to scavenge free radicals [27].

Villaño *et al.* (2005) tested the antioxidant activity of various wine phenolic compounds [33]. Most flavonoids and their glycosides had less antioxidant activity than hydroxybenzoic and hydroxycinnamic acids. Using the DPPH• method, the values decreased in the order Gallic acid > caffeic acid > ferulic acid > resveratrol > *p*-coumaric acid for the same compounds investigated in our study. They found a general correlation between the number of OH groups and the antioxidant power of phenolic compounds, which is in accordance with our results. The highest activities among flavonols corresponded to those with *ortho-*dihydroxylation of the B-ring.

Fukumoto and Mazza (2000) observed an increase in antioxidant activity with an increase of the number of hydroxyl groups for benzoic and cinnamic acid derivates, as well as for flavonols and anthocyanidins [36]. Dziedzic and Hudson (1983) stated that at least two hydroxyl groups are required for the antioxidant activity of phenol acids and found a decrease of antioxidant activity for the glycosylated derivates of quercetin, cyanidin, pelargonidin and peonidin [37].

The positive effect of tyrosinase on the antioxidative capacity of phenolic compounds observed is probably due to autoxidation products emerging after oxidation, catalyzed by tyrosinase. Tyrosinase converts its substrates into highly reactive diquinones, which themselves are powerful oxidants. Subsequent autoxidation, however, finally results in polymeric structures. Melanin biosynthesis is one example of tyrosinase-induced polymerization of a simple phenolic substance, in this case tyrosine. Tyrosinase converts tyrosine into dopaquinone. Afterwards, there are several autocatalyzed intramolecular conversions that lead to intermediates which possess aromatic hydroxyl groups, such as leucodopachrome or 5,6-dihydroxyindole. Further oxidation leads to eumelanin, which itself exhibits a large number of aromatic hydroxyl groups linked to huge delocalized π-systems [38].

Autoxidation of hydroxycinnamates, stilbenes and chalcones after oxidation by tyrosinase is not as well studied as is the case of tyrosine. However, the results of this study suggest that products resulting from resveratrol, polydatin, coumaric acid and phlorizin possess a sufficient number of adjacent aromatic hydroxyl groups to show a stronger scavenging effect than their respective educts. The negligible difference in scavenging activity of ferulic and Gallic acid may be explained by the very low conversion rates regarding these two substrates (see Table 1).

The scavenging effect of all tyrosinase-oxidized hydroxycinnamates used in this study is comparable. This suggests that they form similar oxidation products. The exception mentioned previously in relation to the scavenging activity regarding oxidized and nonoxidized phenolic substrates, in the case of caffeic acid, could be explained by the very strong scavenging effect of caffeic acid itself.

The negative effect of *Polyporus l*accase on the antioxidative power of polyphenols observed cannot simply be explained by the loss of functional hydroxyl groups. The initial enzymatic step of PPO involves the production of an aryloxy radical from the phenolic compound by removal of an electron and a hydrogen ion from the hydroxyl group. The resonance-stabilized intermediates can couple at positions *ortho* or *para* to the hydroxyl group to yield C-C-bound dimers or react further to oligomers and polymers such as melanin. The relative amounts and structures of the final products depend on the initial monomer concentration, chemical reactivity of the intermediates, buffer composition and pH value [15]. The enzymatic coupling products are less water-soluble and often precipitate from solution, rendering them no longer bioactive. We assume that this behavior is a main reason for the negative impact of *Polyporus* laccase on the antioxidant capabilities of the polyphenols in our aqueous test systems. However, laccase-catalyzed oxidations of polyphenols in organic solvents, keeping substrates and products in solution, yield antioxidant materials that are often more powerful than the monomers (as reviewed by Claus *et al.* [13]). This has been shown, for example, for catechin [39] rutin [40] and ferulic acid [26]. 

In theory, tyrosinase and laccase should form similar reaction products when processing substrates with adjacent aromatic hydroxyl groups. The comparable results of the tyrosinase and laccase oxidation products of caffeic, ferulic and Gallic acid in this study support this statement. However, due to the *ortho*-monophenolase activity of tyrosinase, it is likely that very different oxidation products in comparison to laccase are formed, supposing the substrate does not possess adjacent hydroxyl groups. This is the case for resveratrol, polydatin, coumaric acid and phlorizin. Indeed, we observed opposite effects on the antioxidative activity of tyrosinase and laccase oxidation products in these cases. An additional explanation could be the different pH values of reaction buffers used for both PPO. The more acidic pH in the case of laccase may hinder autoxidation of reactive intermediates. Moreover, different concentrations and reaction stabilities of both PPO may generate various products.

## 3. Experimental Section

### 3.1. Chemicals

All reagents were of analytical grade. Polydatin, phlorizin, caffeic acid, ferulic acid, Gallic acid, coumaric acid, DPPH•, DMSO, sodium citrate and *Agaricus* tyrosinase were purchased from Sigma-Aldrich (Taufkirchen, Germany). Resveratrol, malate, tartrate and citric acid were supplied by Roth (Karlsruhe, Germany), and 2,2′-azino-bis(3-ethylbenzothiazoline-6-sulphonic acid (ABTS) from AppliChem (Darmstadt, Germany). The chemiluminescence (CL) dye 8-amino-5-chloro-7-phenylpyridol[3,4-*d*]pyridazine-1,4-(2*H*,3*H*)dione sodium salt (L-012) was obtained from Wako Chemicals (Neuss, Germany), and DMNQ and PMA from Calbiochem/Merck KGaA (Darmstadt, Germany). Hanks’ balanced salt solution was purchased from Gibco Life Technologies GmbH, Eggenstein, Germany. Laccase from *Polyporus pinisitus* was kindly donated by Novo Nordisk Biotech.

### 3.2. Polyphenol Oxidase Activity

#### 3.2.1. Spectrophotometric Determination of Activity

Laccase activity was determined spectrophotometrically by the standard reaction with ABTS. A solution of 10 mg ABTS in tartrate–malate buffer (30 mM) at pH 3.5 was prepared. An amount of 195 µL of the solution was added to 5 µL of enzyme solution in a Rotilab^®^ microtest plate. The formation of the cation radical was detected at 20 °C by measuring the absorbance increase at 420 nm (ε_420_ = 36.000 M^−1^·cm^−1^) using a FLUOstar Omega microplate reader (BMG Labtech GmbH, Ortenberg, Germany). One unit of laccase activity was defined as the amount of enzyme that catalyzed the oxidation of 1 µmol of ABTS in 1 min.

Tyrosinase activity (3130 U/mg) was adopted from the supplier’s specification. One unit was defined as ΔA_280_ of 0.001 per min at pH 6.5 at 25 °C in 3 mL reaction mix containing l-tyrosine.

#### 3.2.2. Oxygen Consumption Assay

The PPO-catalyzed phenol oxidation was monitored by O_2_ consumption with the analyzer NomaSense O_2_ P300 (Nomacorc, Thimister, Belgium) based on oxo-luminescence technology, normally used for noninvasive accurate measurement of oxygen in wine. We established a new and convenient method in addition to the well-known O_2_ electrode [41,42]. Phenolics were solved in 100% ethanol (*v*/*v*) gaining a 50 mM stock solution. The stock solution was diluted to a 5 mM solution in an appropriate air-saturated buffer system for measurement. Experiments with tyrosinase were prepared in citrate buffer (0.1 M pH 5.0) and those with laccase in tartrate–malate buffer (30 mM, pH 3.5). After the O_2_ concentration had been stabilized, laccase (0.7 U/mL) or tyrosinase (313 U/mL) was added to the solution to initiate the reaction.

### 3.3. Determination of Antioxidant Activities of Phenolic Compounds and Their Oxidation Products

#### 3.3.1. Sample Preparation

Two batches containing 5 mM phenolic compound in buffer were prepared as described above for measurements before and after enzymatic oxidation by tyrosinase or laccase. One of the respective batches was mixed with polyphenol oxidase (313 U/mL tyrosinase or 0.7 U/mL laccase) and incubated for 16–24 h at room temperature.

#### 3.3.2. Chemical Assay for Scavenger Activities with the DPPH• Method

The DPPH• method developed by Blois *et al.* [43] was modified referring to the method of Brand-Williams *et al.* [44]. A 0.5 mM stock solution of DPPH• was prepared in 100% methanol, protected from light and stored at 4 °C. The stock solution was diluted freshly for each experiment in methanol until the initial absorbance was approximately 0.62–0.66 at 515 nm and 20 °C (corresponding to a 150 µM working solution). The assay was prepared in microplates in a similar way to the procedure of Fukumoto and Mazza [36]. Twenty-two microliters of sample and 200 µL of DPPH• working solution were added to 96-well flat-bottom Rotilab^®^ microtest plates from Roth (Karlsruhe, Germany). Samples were prepared in triplicate. The plate was covered, kept in the dark at 20 °C and read every 30 s for 30 min using a 515 nm filter. A methanol blank for each sample was also measured. The initial absorbance was determined by mixing 200 µL of the DPPH• working solution with 22 µL buffer and set as 100% available DPPH•. The reduction of absorbance was converted as a percentage of the remaining DPPH•.

#### 3.3.3. Biological Antioxidant Assay Using Cell Cultures

Superoxide was detected with the luminol analog L-012 in cell culture systems [45]. Human endothelial EA.hy 926 cells [46] and human monocytic THP1 cells [47] were used for this experiment. In order to stimulate superoxide production, EA.hy cells were incubated with DMNQ (10 μM) and THP-1 cells were treated with PMA (10 nM), an activator of protein kinase C (PKC). In brief, cells were incubated with phenolic substances (5 µM) in Hanks’ balanced salt solution for 15 min at 37 °C. Then, L-012 (100 µM) and DMNQ (for EA.hy cells) or PMA (for THP-1 cells) were added. The phenolic compounds were applied with DMSO in the same concentration as the solvent control. The negative control, which showed no stimulation of superoxide production, contained no phenolic compounds, DMNQ or PMA (all substituted with DMSO). Superoxide-induced chemiluminescence (CL) was determined after 30 min dark adaptation using a Centro LB960 plate luminometer (Berthold Technologies, Bad Wildbad, Germany).

## 4. Conclusions

PPO show a strong influence on the antioxidative capacity of various phenolic compounds. This effect was observed chemically with the classical DPPH• assay, as well as in human cell lines. Tyrosinase from *Agaricus bisporus* tends to increase the antioxidative capacity of phenolic substances, whereas laccase from *Polyporus pinisitus* has the opposite effect. These differences may be explained by the specific monohydroxylase activity of tyrosinase, which is absent in laccase. However, the nature of the products and elucidation of the reaction mechanisms afford further investigations. Nevertheless, the results of our study may be interesting for enzymatic synthesis of new bioactive polyphenols.
